# Host factors influence Barrett’s carcinogenesis: findings from a mouse gastroduodenal reflux model

**DOI:** 10.1007/s10388-019-00660-5

**Published:** 2019-02-21

**Authors:** Shunpei Kanai, Ken-ichi Mukaisho, Saori Yoshida, Naoko Taniura, Hiroyuki Sugihara

**Affiliations:** Division of Molecular and Diagnostic Pathology, Department of Pathology, Shiga University of Medical Science, Seta-tsukinowa-cho, Otsu, Shiga 520-2192 Japan

**Keywords:** Barrett’s esophagus, Carcinogenesis, Bile acids, Taurine conjugate, Mouse

## Abstract

**Background:**

Rat gastroduodenal reflux models have been used for analyzing Barrett’s carcinogenesis. Mice seem to be more useful than rats for studies targeting genes.

**Methods:**

We induced gastroduodenal contents reflux by esophagojejunostomy using C57BL/6J mice. Mice were divided into a standard diet and high-fat diet groups and kept for 60 weeks. Bile was sampled from the gallbladder to analyze bile acid fractions, and the esophagus was removed for a histological investigation. Human esophagogastric junction adenocarcinoma cells (OE19) were exposed to taurocholic acid (TCA), after which cell proliferative activity was measured. Rat esophageal cancer cell lines, ESCC-DR and ESCC-DRtca with higher malignant potential induced by continuous TCA exposure, were used to perform comprehensive genetic analysis (CGH).

**Results:**

Barrett’s epithelium onset occurred in all mice, and no differences in histological changes were noted between the standard diet and high-fat diet groups. However, no development of adenocarcinoma was noted. Most of the mouse bile acid was taurine conjugates. In the experiment using OE-19 cells, TCA promotes cell proliferation in a dose-dependent manner. Array CGH analysis revealed a large number of chromosomal abnormalities in the ESCC-DR, in addition to genetic abnormalities such as in the UGT2B gene, the substrate of which is bile acid. TCA administration resulted in more chromosomal abnormalities being detected.

**Conclusions:**

We showed the effects of TCA in cancer progression in vitro. However, Barrett’s adenocarcinoma onset rates differ between mice and rats despite undergoing similar reflux stimulation including taurine-conjugated bile acids being detected in mouse bile juice. These results suggest that host factors seem to influence Barrett’s carcinogenesis.

## Background

Gastroesophageal reflux is a significant risk factor for the onset of esophageal adenocarcinoma. In Western countries, the incidence of gastroesophageal reflux disease (GERD) is increasing as a result of a rapid increase in obesity associated with high-fat diets [1]. Adenocarcinoma is now accounting for over 60% of cases of esophageal cancer overall in the USA [2]. Barrett’s epithelium, which indicates the presence of columnar metaplasia in the esophagus, has been reported to be a precancerous lesion suggestive of esophageal adenocarcinoma [3].

We have previously surgically induced the development of adenocarcinoma from Barrett’s epithelium in rats [4–6]. We have also reported that by providing such reflux model animals with a high-fat diet filled with beef tallow, the incidence of Barrett’s epithelium increased, resulting in a higher incidence of esophageal tumors [5]. Research, utilizing genetic information, remains essential in gaining a deeper understanding of the onset process of Barrett’s epithelium leading to adenocarcinoma. For this purpose, mice appear to be more useful than rats, as the genetic background of mice has been clarified in more detail than that of rats. In this study, we examined the histological changes in mice reflux modes using additional immunohistochemical stainings of CDX2, PDX1, and CK7, because CDX2 is a homeobox domain-containing transcription factor that is important in the development and differentiation of the intestines and it is widely accepted that CDX2 plays key roles on differentiation into the intestinal-type cell characteristic of Barrett’s esophagus [7, 8]. PDX1 is reported to be a marker of a true Barrett’s epithelium [9]. The expression of CK7 might help to explain the pathological, reflux-related nature of columnar-lined esophagus, as an aberrant expression in a very early stage of the multistep Barrett’s esophagus progression [10]. Recently, Jiang used CK7 as a marker of Barrett’s esophagus [11].

Mouse models have been previously proposed to research the onset process of Barrett’s epithelium. Accordingly, the present study aimed to establish a duodenal reflux model of Barrett’s carcinogenesis using mice. We also need to compare the effects of gastroduodenal reflux on esophagus between mice and rats. Thus, we utilized the same techniques used in the past to construct rat reflux models on mice [5, 8]. However, the onset rates of Barrett’s epithelium and adenocarcinoma in mouse models have been extremely low compared to rat models [9, 12]. To increase the incidence of Barrett’s epithelium and adenocarcinoma in the present study, we provided mice with a high-fat diet after performing surgery [5] and we also extended the postoperative period until killing, compared to the study using rat reflux models. However, these treatments did not result in tumor onset from Barrett’s epithelium in this study. To verify these results, we used taurocholic acid (TCA), which has been reported to be actively involved in the onset of Barrett’s epithelium and adenocarcinoma, to perform an in vitro investigation of cell proliferative activity using human esophagogastric junction adenocarcinoma cell line (OE19). It has been reported that the American prevalences of esophageal adenocarcinoma and esophagogastric junction adenocarcinoma have increased in tandem, because esophagogastric junction adenocarcinoma is related to GERD [13]. We also used array-based comparative genomic hybridization (CGH) to perform a comprehensive analysis of ESCC-DR cells established from the intrapleural metastasis focus of squamous cell carcinoma that developed in a rat reflux model and ESCC-DRtca cells with higher malignant potential induced by continuous TCA exposure [14]. In the present study, we reinvestigate the effects of bile acid and discuss factors in Barrett’s carcinogenesis.

## Materials and methods

This study confirmed to the ethical regulations on the use of experimental animals, and all experiments were conducted based on the animal experiment guidelines of the Research Center for Animal Life Science of the Shiga University of Medical Science (experiment approval no.: 2016-5-51).

### Laboratory animals and the feed

We used 8-week-old male C57BL/6J mice to construct a duodenal reflux model and sham surgery group. These mice were divided into two groups according to the diet. The feed administered was the same as that used in a previous rat reflux model [5]. The feed used was a low-fat diet of soybean oil derived from soybeans (a standard diet group: CE-2, containing 4.8% soybean oil) and a high-fat diet made of beef tallow (a high-fat diet group: Quick Fat, containing 15.3% beef tallow). Both were purchased from CLEA Japan, Inc. (Osaka, Japan).

### Construction of duodenal reflux model and a sham surgery group

**Fig. 1:**
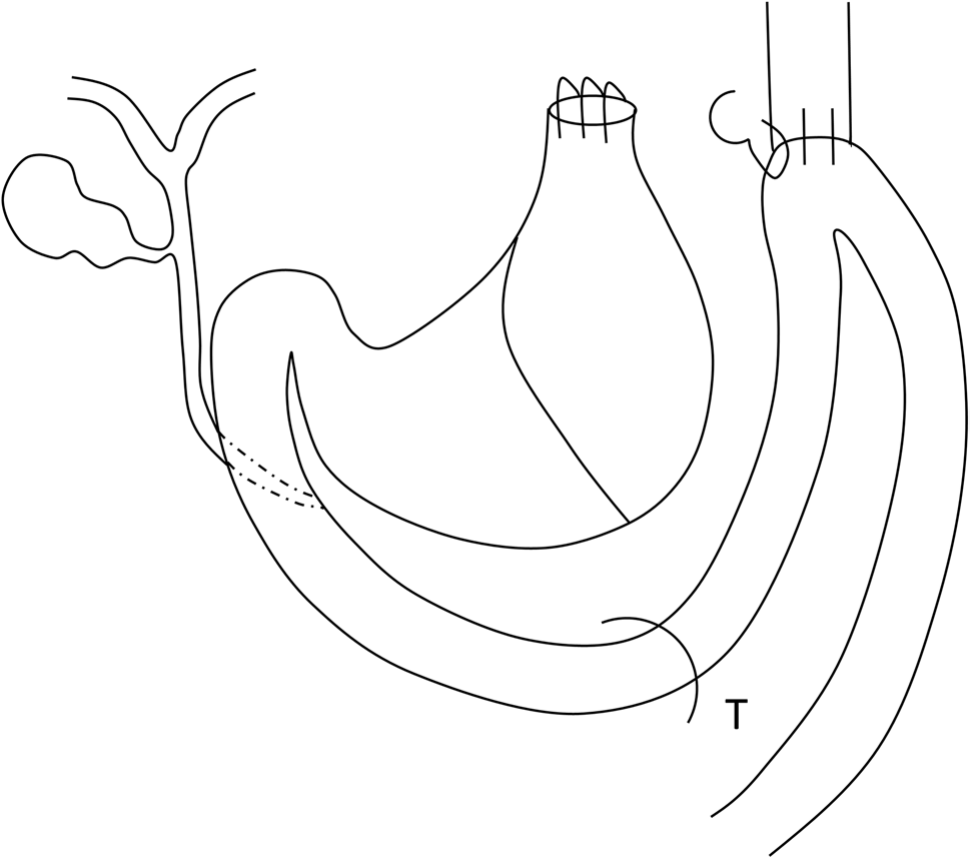
Mouse reflux model. *T* Treitz ligament. We performed a gastroduodenal contents reflux surgery by esophagojejunostomy without gastrectomy. There are anatomical differences in gallbladder between rat and mouse. While mice have a gallbladder, rats do not

As a preoperative preparation, the subjects were made to fast for 24 h before surgery, only ingesting water. All procedures were performed with the subjects under inhalation anesthesia with isoflurane. The surgical method used was almost identical to techniques previously used to construct rat duodenal reflux models [5, 8]. First, the E–G junction was transected after laparotomy, and the gastric stump was closed with continuous sutures using 8-0 nylon (CROWNJUN, Chiba, Japan). End-side anastomosis was performed between the esophageal cut end and the upper jejunum located approximately 2 cm distally from the pylorus ring. We added one more serosal suture to the esophagojejunal anastomosis (Fig. 1). Physiological saline solution was injected into the peritoneal cavity to prevent adhesion and as postoperative fluid replenishment, and the wound was closed. For the construction of the sham surgery group, the same pre- and postoperative preparation was implemented for the reflux model. Blunt operations, such as gripping the organs in the abdominal cavity, were performed after laparotomy.

### Bile and esophageal tissue collection

At 60 weeks postoperatively, thoracotomy and laparotomy were performed under inhalation anesthesia with isoflurane. The gallbladder was perforated with a 29-G needle 0.5-mL syringe (Terumo Corporation, Tokyo, Japan), and bile was suctioned out. The collected bile was stored at − 80 °C until analysis. Bile acid analysis was performed by Junshin Clinic Bile Acid Institute (Tokyo, Japan). The anastomosed upper jejunum and esophagus were collected together. The removed samples were fixated with 10% buffered formalin, and paraffin blocks were prepared. The paraffin blocks were cut into 2-µm slices, and hematoxylin and eosin (HE) staining was performed for histological investigation.

### Immunostaining

The 2-µm slice specimens created from paraffin blocks underwent immunostaining with primary antibodies of CK7 (dilution time is 250:1, cat. #ab9021, Abcam, Tokyo, Japan), PDX1 (500:1, cat. #ab9021, Abcam) and CDX2 (250:1, cat. #ab76541, Abcam). Histofine MAX-PO (Nichirei, Tokyo, Japan), which is specialized for use on mouse tissue, was used to activate the antigens with heat treatment, and visualization was then performed using DAB (Nichirei).

### Effects of TCA exposure on cell proliferation

The human esophagogastric junction cancer cell line (OE-19) was purchased from Summit Pharmaceuticals International Corporation (Tokyo, Japan). The cells were subcultured in an RPMI 1640 (Nacalai Tesque Co., Kyoto, Japan) medium adjusted so that the antibiotic–antimycotic solution (Life Technologies Co., CA, USA) and FBS were 1% and 10%, respectively. OE-19 cells were then seeded in a black-walled 96-well plate (Thermo Scientific Nunc; Thermo Fisher Scientific, MA, USA) at 1 × 10^4^ cells/well. After 24 h, the specimens were washed with D-PBS (Nacalai Tesque Co., Kyoto, Japan) and then with modified EPM2 (AthenaES, MD, USA). Next, specimens were switched to a modified EPM2 medium with each concentration of TCA (Sigma, MO, USA) and cultured at 37 °C with 5% CO_2_. The added TCA concentrations were 0, 50, 100, 500, and 1000 μM. 48 h after adding the reagent, washing was performed twice with D-PBS. Then, D-PBS was added at 50 μL/well. Then, 4 μM Calcein-AM (Dojindo Laboratories, Kumamoto, Japan) was added to this at 50 μL/well and cultured for 1 h at 37 °C. The plate was removed and fluorescence intensity at 515 nm was measured with excitation rays at 490 nm.

### Array CGH analysis

1. Genetic analysis of esophageal cancer cells developed in a rat reflux model
  To identify genes involved in cancer onset in a rat reflux model, while we did not have any rat Barrett’s adenocarcinoma cell line, we performed comprehensive genetic analysis of the ESCC-DR cells with normal rat esophageal epithelium as the reference.
2. Genetic analysis of tumor expansion resulting from TCA administration
  To identify the genetic abnormalities caused by tumor expansion resulting from TCA administration, we performed comprehensive genetic analysis with ESCC-DR as the reference and ESCC-DRtca as the test sample.

In both of the above analyses, array CGH analysis was performed using Rat Genome CGH Microarray, 4 × 180K (Agilent, CA, US). An analysis was performed according to the manufacturer’s instructions using DNA Chip Research Inc. (Tokyo, Japan). Copy number gains and losses were defined as changes in the logarithm to the base 2 of the tumor to reference signal intensity ratio (T/R) > 0.3219 and < −0.3219, respectively.

### Statistical analysis

To detect statistically significant differences in the nominal variables of the two groups for animal model experimental data and bile acid fraction analysis, we used Fisher’s exact test. For continuous variables, we used the Mann–Whitney *U* test. Figures were displayed as median values, and a *P* value < 0.05 indicated statistical significance.

## Results

### Postoperative changes in mouse weight

There were 18 surviving mice in the standard diet group, 20 surviving mice in the high-fat diet group, and 5 mice each in the standard diet and high-fat diet groups that underwent sham surgery. In the mouse duodenal reflux model, the median weight was 27.2 g in the standard diet group and 33.2 g in the high-fat diet group, indicating that subjects were significantly heavier in the high-fat diet group (*P* value < 0.05). In the sham surgery group, the median weight was 37.1 g in the CE2 intake group and 48.2 g in the high-fat diet group, indicating that subjects were significantly heavier in the high-fat diet group (*P* value < 0.05).

### Barrett’s epithelium development

**Fig. 2:**
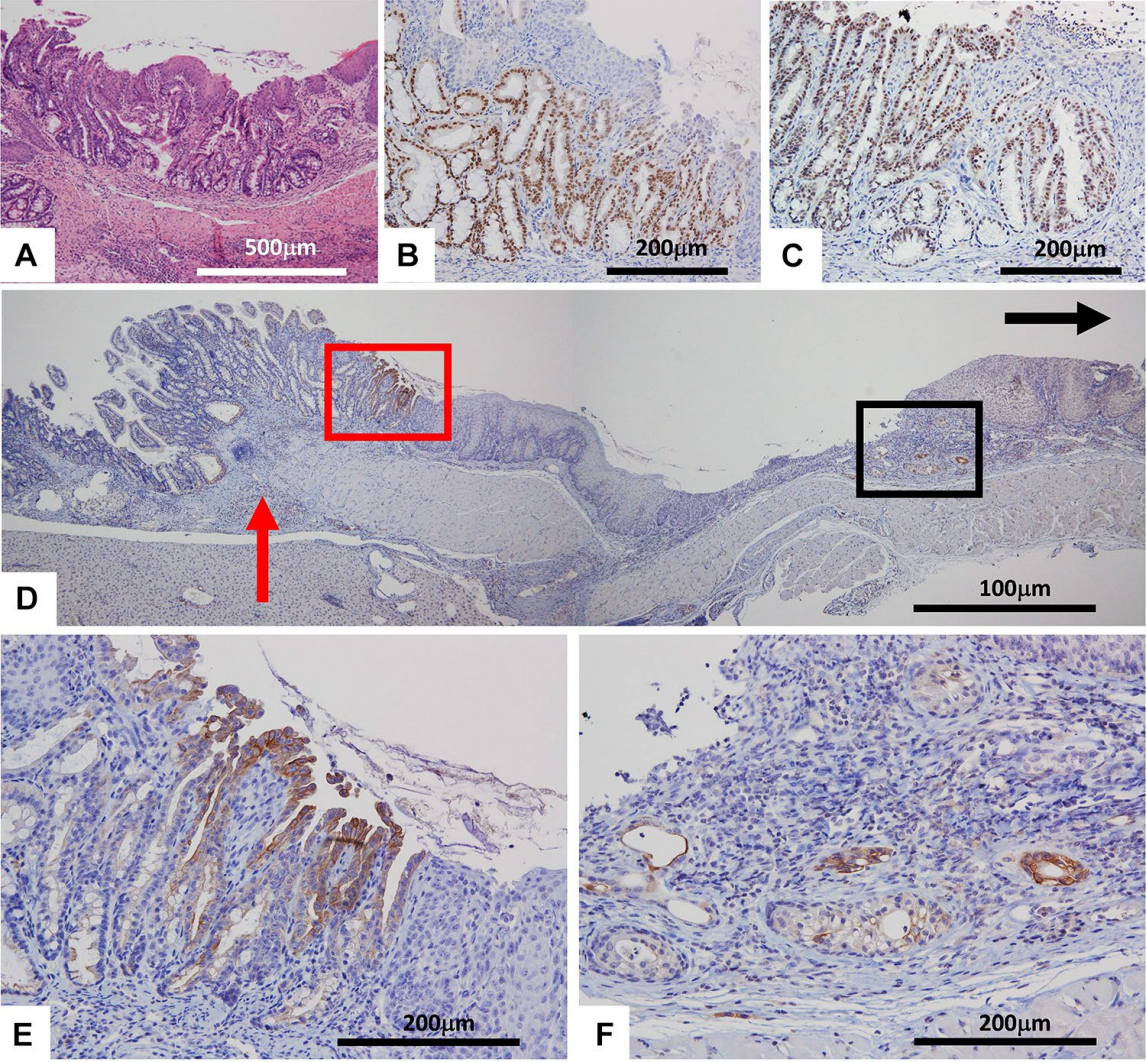
Immunohistochemical stainings of Barrett’s epithelium. **a** HE staining, **b** CDX2, **c** PDX1, **d** CK7, **e** Higher magnification of red square in (**d**). **f** Higher magnification of black square of (**d**). Black arrow and red arrow in (**d**) indicate oral site and the anastomotic site, respectively. The Barrett’s epithelium was mostly developed near the esophagojejunal anastomosis (**a**, **d** and **e**). The Barrett’s epithelium strongly expressed CDX2 in (**b**), PDX2 in (**c**), and CK7 in the red square of (**d, e**). Small numbers of CK7-positive cells were noted at sites distant from the anastomotic site in each group in the black square of (**d, f**)

We were able to confirm that Barrett’s epithelium developed in all subjects in the mouse duodenal reflux model near the esophagojejunal anastomosis (Fig. 2a). No marked differences of histological results were observed between the two groups. All Barrett’s epithelium had intestinal metaplasia positive for CDX2 (Fig. 2b). PDX1 is also strongly positive for Barrett’s metaplasia (Fig. 2c). There were no remarkable differences in the positivity of PDX1 between the two groups. Although CK7 was negative for proper jejunal mucosa and squamous epithelium, Barrett’s epithelium was positive for CK7 (in the red square of Fig. 2d, e). Some, albeit small numbers of, CK7-positive cells were noted at sites distant from the anastomotic site in each group (in the black square of Fig. 2d, f). While these cells were negative for CDX2, results suggested these cells are in a very early stage of the multistep Barrett’s esophagus progression and might be in a stage prior to direct metaplasia in the columnar epithelium. These CK7-positive cells seemed to be developed from the basal layer of the esophagus-stratified squamous epithelium in the regenerative process. Meanwhile, no development of Barrett’s epithelium was noted in the sham surgery groups. Moreover, no onset of Barrett’s adenocarcinoma was observed in all animals.

### Bile acid analysis

A sufficient amount of bile for bile acid fraction analysis was suctioned from perforations in the gallbladders in five reflux animals in the normal diet group and five reflux animals in the high-fat diet group. Of the 107 items investigated in detail, we showed the 10 items that were measurable in all of the above 10 samples and compared them between the two groups (Table 1). T-α-MCA was significantly increased in the high-fat intake group (*P* value = 0.046). No other significant differences were noted between the groups for bile acid (Table 1).

**Table 1:** Bile acid composition (mmol/L) of bile juice aspirated from gallbladder of the reflux models in each group

	60 W (reflux models)		
	S (mmol/L)	H (mmol/L)	p value
Taurocholic acid (TCA)	55.6	58.1	0.841
Taurochenodeoxycholic acid (TCDCA)	1.1	1.1	1
Taurodeoxycholic acid (TDCA)	0.7	1.1	0.916
Taurohyocholic acid (THCA)	0.1	0.1	0.424
Taurohyodeoxycholic acid (THDCA)	4.3	8.1	0.841
Tauro-α-muricholic acid (T-α-MCA)	2.8	4.2	0.0459*
Tauro-β-muricholic acid (T-β-MCA)	20.3	25.3	0.151
β-muricholic acid (β-MCA)	0.1	0.1	0.699
Tauro-ω-muricholic acid (T-ω-MCA)	5.3	6.7	0.421
Total bile acids (TBA)	98.7	100.6	0.548

Values are expressed as median

*H* high-fat group fed a high cow-fat diet (Quick Fat), *S* standard diet group fed a soybean oil diet (CE-2)

*Significant difference, *P* < 0.05

### Effects of bile acid exposure on cell proliferation

**Fig. 3:**
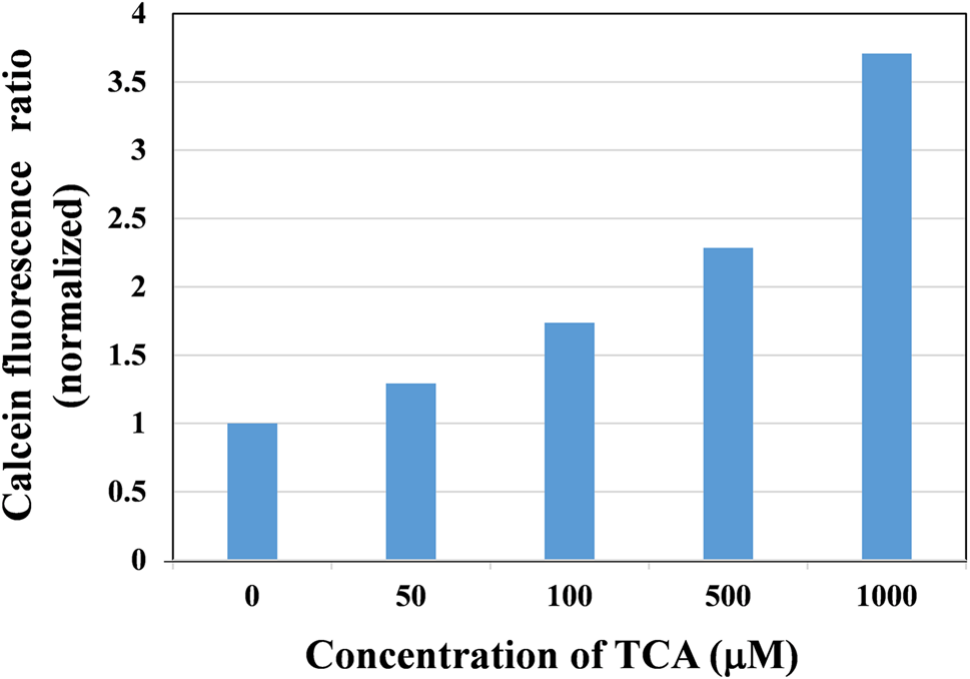
The effects of TCA administration on cell proliferation in OE19 cells. TCA promotes cell proliferation in a dose-dependent manner

TCA administration resulted in increases in cell proliferative activity in OE19 in a dose-dependent manner (Fig. 3).

### Genetic abnormalities in ESCC-DR

Of the genes, such as UGT2B, that were amplified by array CGH analysis of ESCC-DR, the ten with the highest amplification rates were ranked in order (only genes for which the gene name has been clarified) and are shown in Table 2.

**Table 2:** Up-regulated genes in ESCC-DR obtained by arrayCGH analysis

	UCSC position/search	Name of gene	Ratio
1	chr9: 110301814–110301873	MYOM1	6.22
2	chr10: 87353525–87353584	IKZF3	5.98
3	chr3: 22733900–22733959	LRP1B	5.86
4	chr17: 14874142–14874201	CATSPER3	5.55
5	chr6: 101948867–101948926	RDH11	5.40
6	chr3: 47830793–47830852	CSRNP3	5.32
7	chr14: 22158617–22158676	UGT2B	4.70
8	chr2: 21149053–21149112	ATG10	4.69
9	chr2: 191559460–191559519	RNF115	4.05
10	chr9: 71822103–71822162	SMARCAL1	3.98

### Comparison of ESCC-DR and ESCC-DRtca

**Fig. 4:**
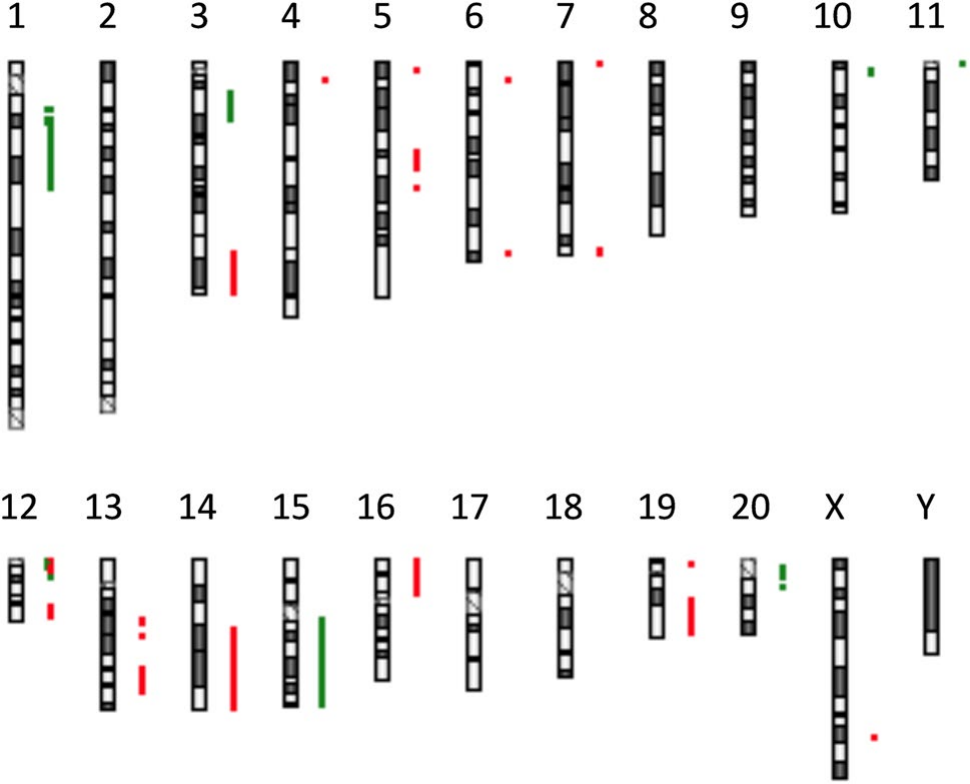
Comparison of ESCC-DR and ESCC-DRtca by array CGH. Gains and losses are indicated with red and green, respectively. We performed comprehensive genetic analysis with ESCC-DR as the control and ESCC-DRtca as the test sample. Many chromosomal abnormalities were detected

Comparison of ESCC-DR and ESCC-DRtca revealed that when tumor expansion resulted from TCA administration, many chromosomal abnormalities developed (Fig. 4).

## Discussion

In the mouse reflux model, cancer onset from Barrett’s epithelium was not observed even when a high-fat diet was administered. We were only able to confirm Barrett’s epithelium near the anastomotic site. The Barrett’s epithelium was positive for PDX-1 in this study. It has been reported that PDX-1 is usually expressed in the gastric antrum, duodenum, and pancreas, and is absent in the esophagus, gastric fundus, and jejunum. On the other hand, PDX-1 is initially negative in the jejunum. Gastric intestinal metaplasia was reported to be positive for PDX-1, suggesting duodenal metaplasia rather than general intestinal metaplasia [15]. Furthermore, we did not observe any columnar epithelium metaplasia located extensively throughout the proximal side of the esophagus and far from the anastomotic site as was the case for a rat reflux model [16].

The fact that Barrett’s adenocarcinoma onset rates and histogenesis of Barrett’s epithelium differ between mice and rats despite undergoing similar reflux stimulation may have been due to the following two reasons: First, it could have been due to anatomical differences between rat and mouse gallbladders. In rat reflux models without a gallbladder, bile reflux occurs continuously into the esophagus together with duodenal fluid. However, in a mouse reflux model with a gallbladder, there appears to be no continuous bile reflux into the esophagus even if the upper jejunum is surgically anastomosed to the esophagus. In human cases, it has been reported that esophageal adenocarcinoma incidence increases after gallbladder extraction [17]. This suggests that the presence of the gallbladder may affect the onset of adenocarcinoma. The second possible reason is that bile acid constitution and bile acid may have different effects on esophageal tissue in rats and mice. Human bile acid is said to contain a large proportion of glycine-conjugated bile acid with glycine conjugates-to-taurine conjugates constitution at 3:1 [18]. Nehra et al. have reported that in cases that exhibit Barrett’s epithelium and esophagitis onset, taurine conjugates are dominant in the esophagus [19]. We previously reported that an increased proportion of taurine conjugates, due to a high-fat diet, affected Barrett’s epithelium formation and atypical epithelium onset rates in the rat reflux model [5], and chronic exposure to TCA caused tumor progression [14]. Accordingly, in our study, we performed an additional in vitro experiment to confirm how TCA affects tumor progression. Research using esophagogastric junction adenocarcinoma cell line (OE-19) has indicated that TCA causes more increases in cell proliferative activity. In vitro, the other experiments also indicated that taurine-conjugated bile acids activate Src, EGFR, and ERK, thereby causing colorectal cancer cells to proliferate [20]. The present study also showed that more chromosomal abnormalities developed in ESCC-DRtca than ESCC-DR. In this study, bile acid fraction analysis of mouse gallbladder bile indicated that most were taurine conjugates, and the administration of a high-fat diet led to significant increases in T-α-MCA. However, Barrett’s epithelium only developed near the esophagus–jejunum anastomotic site, and no cases of adenocarcinoma were noted. Terabe et al. performed esophagogastrojejunostomy (side-to-side) without total gastrectomy [9]. They reported that these model developed metaplasia and dysplasia more frequently than the model of esophageal separation and esophagojejunostomy (end-to-side) similar to our model in the present study [9]. The model may be superior to our reflux model on the point of inducing both gastric acid and bile acid reflux to occur. For a mice model in the future, we might use the model of esophagogastrojejunostomy. However, even in this model no adenocarcinoma developed in the previous study [9]. These findings suggest that host factors may influence Barrett’s carcinogenesis.

As one of the host factors affected in Barrett’s carcinogenesis, we focused on the fact that UGT2B amplification was noted in the analysis of genetic abnormalities in the ESCC-DR cell line. Bile acid is an essential substrate for UGT2B, and one can easily see that UGT2B is involved in cancer onset in reflux models with significant exposure to bile acid. It has been confirmed that UGT is present in not only the liver, but also the small intestine, kidneys, brain, and esophagus [21, 22]. UGT2B is present in esophageal squamous cell carcinoma, with some recent reports indicating that it is related to prostate cancer and UGT2B genetic polymorphisms [23, 24]. Accordingly, genetic polymorphisms between UGT2B specimens might also be actively involved in the metabolism of carcinogens in the digestive tract. We previously reported that endogenous nitrosated bile acid conjugates derived from duodenal content reflux have mutagenicity and the *N*-nitroso bile acid is actively involved in Barrett’s carcinogenesis [25–27]. Although we did not compare the UGT2B polymorphisms between rats and mice, it might be that UGT expression in the esophagus and small intestine differs between mice and rats. This suggests that the detoxification of nitroso compounds produced from bile and gastric acid and the metabolism of other carcinogens may also differ between rats and mice. An epidemiological study on humans found that Barrett’s adenocarcinoma incidence has been rapidly increasing mainly among white men, demonstrating clear racial- and gender-related differences in the incidence of Barrett’s adenocarcinoma [28]. These findings suggest that the effects of bile acid and other carcinogens on esophageal mucosa may differ depending on host factors such as UGT2B genetic polymorphisms.

We understand that there is a limitation in the present study. It is very challenging to say something about Barrett’s carcinogenesis from the present results based on a mice model, because we failed to establish the mouse model for inducing Barrett’s adenocarcinoma. We found that most of the mouse bile acid was taurine conjugates, and we confirmed the effects of TCA in cancer progression in vitro using OE-19, ESCC-DR, and ESCC-DRtca. If we had obtained mice Barrett’s adenocarcinoma cells, we might have shown the validity of adapting the data of CGH and genetic polymorphism on rat squamous cell line to mouse Barrett’s carcinogenesis more clearly. However, we can say that Barrett’s adenocarcinoma onset rates differ between mice and rats despite undergoing similar reflux stimulation, and that host factors seem to influence Barrett’s carcinogenesis. Although we had continued to use rat reflux models to perform research focusing on risk factors for Barrett’s carcinogenesis until now, host factor effects also need to be taken into consideration in the future. One factor for this appears to be the presence of the UGT-2B gene and its genetic polymorphisms, which may be involved in host physiological reactions to bile acid irritation.

## References

[1] Edelstein ZR, Farrow DC, Bronner MP (2007). Central adiposity and risk of Barrett’s esophagus. Gastroenterology.

[2] Everhart JE, Ruhl CE (2009). Burden of digestive diseases in the United States part I : overall and upper gastrointestinal diseases. Gastroenterology.

[3] Spechler SJ, Fitzgerald RC, Prasad GA (2010). History, molecular mechanisms, and endoscopic treatment of Barrett’s esophagus. Gastroenterology.

[4] Kumagai H, Mukaisho K, Sugihara H (2003). Cell kinetic study on histogenesis of Barrett’s esophagus using rat reflux model. Scand J Gastroenterol.

[5] Chen KH, Mukaisho K, Sugihara H (2007). High animal-fat intake changes the bile-acid composition of bile juice and enhances the development of Barrett’s esophagus and esophageal adenocarcinoma in a rat duodenal-contents reflux model. Cancer Sci.

[6] Chen KH, Mukaisho K, Ling ZQ (2007). Association between duodenal contents reflux and squamous cell carcinoma–establishment of an esophageal cancer cell line derived from the metastatic tumor in a rat reflux model. Anticancer Res.

[7] Souza RF, Krishnan K, Spechler SJ (2008). Acid, bile, and CDX: the ABCs of making Barrett’s metaplasia. Am J Physiol Gastrointest Liver Physiol.

[8] Tatsuta T, Mukaisho K, Sugihara H (2005). Expression of Cdx2 in early GRCL of Barrett’s esophagus induced in rats by duodenal reflux. Dig Dis Sci.

[9] Terabe F, Aikou S, Aida J (2017). Columnar metaplasia in three types of surgical mouse models of esophageal reflux. Cell Mol Gastroenterol Hepatol..

[10] Cabibi D, Fiorentino E, Pantuso G (2009). Keratin 7 expression as an early marker of reflux-related columnar mucosa without intestinal metaplasia in the esophagus. Med Sci Monit..

[11] Jiang M, Li H, Zhang Y (2017). Transitional basal cells at the squamous-columnar junction generate Barrett’s oesophagus. Nature.

[12] Aikou S, Aida J, Takubo K (2013). Columnar metaplasia in a surgical mouse model of gastro-esophageal reflux disease is not derived from bone marrow-derived cell. Cancer Sci.

[13] Devesa SS, Blot WJ, Fraumeni JF (1998). Changing patterns in the incidence of esophageal and gastric carcinoma in the United States. Cancer.

[14] Sato S, Yamamoto H, Mukaisho K (2014). Continuous taurocholic acid exposure promotes esophageal squamous cell carcinoma progression due to reduced cell loss resulting from enhanced vascular development. PLoS One.

[15] Leys CM, Nomura S, Rudzinski E (2006). Expression of Pdx-1 in human gastric metaplasia and gastric adenocarcinoma. Hum Pathol.

[16] Kushima R, Mukaisho K, Takemura S (2013). Barrett’s esophagus: analyses from human and experimental animal studies. Pathologe.

[17] Freedman J, Ye W, Näslund E (2001). Association between cholecystectomy and adenocarcinoma of the esophagus. Gastroenterology.

[18] Stamp DH (2002). Three hypotheses linking bile to carcinogenesis in the gastrointestinal tract: certain bile salts have properties that may be used to complement chemotherapy. Med Hypotheses.

[19] Nehra D, Howell P, Williams CP (1999). Toxic bile acids in gastro-oesophageal reflux disease: influence of gastric acidity. Gut.

[20] Dossa AY, Escobar O, Golden J (2016). Bile acids regulate intestinal cell proliferation by modulating EGFR and FXR signaling. Am J Physiol Gastrointest Liver Physiol.

[21] Mackenzie PI, Owens IS, Burchell B (1997). The UDP glycosyltransferase gene superfamily: recommended nomenclature update based on evolutionary divergence. Pharmacogenetics..

[22] Strassburg CP, Strassburg A, Nguyen N (1999). Regulation and function of family 1 and family 2 UDP-glucuronosyltransferase genes (UGT1A, UGT2B) in human oesophagus. Biochem J..

[23] Grant DJ, Hoyo C, Oliver SD (2013). Association of uridine diphosphate-glucuronosyltransferase 2B gene variants with serum glucuronide levels and prostate cancer risk. Genet Test Mol Biomark.

[24] Grant DJ, Chen Z, Howard LE (2017). UDP-glucuronosyltransferases and biochemical recurrence in prostate cancer progression. BMC Cancer..

[25] Mukaisho K, Nakayama T, Hagiwara T (2015). Two distinct etiologies of gastric cardia adenocarcinoma: interactions among pH, Helicobacter pylori, and bile acids. Front Microbiol..

[26] Kumagai H, Mukaisho K, Sugihara H (2004). Thioproline inhibits development of esophageal adenocarcinoma induced by gastroduodenal reflux in rats. Carcinogenesis.

[27] Terasaki M, Totsuka Y, Nishimura K (2008). Detection of endogenous DNA adducts, O-carboxymethyl-2′-deoxyguanosine and 3-ethanesulfonic acid-2′-deoxycytidine, in the rat stomach after duodenal reflux. Cancer Sci.

[28] Cossentino MJ, Wong RK (2003). Barrettʼs esophagus and risk of esophageal adenocarcinoma. Semin Gastrointest Dis.

